# Biomimetic Super Anti-Wetting Coatings from Natural Materials: Superamphiphobic Coatings Based on Nanoclays

**DOI:** 10.1038/s41598-018-30586-4

**Published:** 2018-08-13

**Authors:** Jie Dong, Junping Zhang

**Affiliations:** 1 Key Laboratory of Clay Mineral Applied Research of Gansu Province, and State Key Laboratory of Solid Lubrication, Lanzhou Institute of Chemical Physics, Chinese Academy of Sciences, 730000 Lanzhou, P.R. China; 2 University of Chinese Academy of Sciences, 100049 Beijing, P.R. China

## Abstract

Superamphiphobic coatings (SAPCs) that resist wetting of water and low-surface-tension liquids have generated widespread attention in academia, but are very challenging to invent. Liquid adhesion, low stability, complicated and expensive preparation methods are the typical characteristics of SAPCs, which seriously hinder real-world applications of SAPCs. Here, we report a comprehensive study about preparation of SAPCs from abundant nanoclays with fibrous, plate-like and porous microstructures. The SAPCs are prepared simply by hydrolytic condensation of silanes in the presence of nanoclays, followed by spray-coating the as-formed suspensions onto substrates. The SAPCs feature high superamphiphobicity for various liquids down to a surface tension of 23.8 mN m^−1^ (*n*-decane), and high mechanical, chemical and thermal stability. The superamphiphobicity and stability depend on microscale and nanoscale surface morphology of the coatings, which are controllable by the microstructures of nanoclays and their acid activation. The fibrous nanoclays with moderate aspect ratio like palygorskite are the most suitable building blocks for the preparation of SAPCs by effectively forming the reentrant surface morphology. We believe that the findings will promote the progress of SAPCs, and pave the way for the development of clay-based super anti-wetting coatings.

## Introduction

Superamphiphobic coatings (SAPCs) that resist wetting of water and low-surface-tension liquids are of great interest in academia and industry^1–3^. However, different from superhydrophobic coatings, preparation of SAPCs is very challenging owing to the very low surface tension of some liquids, for example, *n*-decane (23.8 mN m^−1^) in contrast with water (72.8 mN m^−1^)^4–7^. Combining a microstructured surface and a low-surface-energy material is difficult to form SAPCs, especially those with low sliding angles (SAs)^8–10^. The interaction between the coatings and the liquids should be sufficiently weak in order to have low SAs for such liquids^11^. So far, only a few groups have reported SAPCs that organic liquids could fall down with low SAs by designing special surface morphology (e.g., reentrant structure, overhang structure and silicone nanofilaments) and by using fluoroPOSS with very low surface energy^11–14^. Tuteja *et al*. demonstrated that the combination of reentrant structure and fluoroPOSS could form extremely non-wetting surfaces to water and organic liquids^12,15^. Liu *et al*. designed doubly reentrant structures in order to prepare SAPCs superrepellent to completely wetting liquids^16^. However, the low mechanical stability of most SAPCs is a key issue in the field^17,18^. Also, the methods for preparation of SAPCs are often complicated and expensive, which limits their applications. So, we invented mechanically durable and self-healing SAPCs using nanorod-like palygorskite (PAL)^18^.

Clay-based super anti-wetting coatings, superhydrophobic or superamphiphobic, are receiving growing attention since 2013^18–20^. Different from most of the reported super anti-wetting coatings, the micro-/nanostructures of the clay-based ones are built with natural nanomaterials^21^. Clays are natural nanomaterials with diverse microstructures^22^, such as nano-platelets and nanofibers, and thus are ideal building blocks for constructing micro-/nanostructures of super anti-wetting coatings^23,24^. Also, clays have many advantages like abundant in nature, low cost and environmentally friendly^22^. There are many kinds of clays on the earth, such as kaolin^25^, montmorillonite (MMT)^26^, PAL^27^, sepiolite^28^ and mica^29^. Clays have been widely used in various fields, e.g., polymer reinforcement^30,31^, superabsorbent composites^32^, dye adsorption^28^, tissue regeneration^33^ and so on. We have prepared superhydrophobic coatings in 2013^19^ and SAPCs in 2016^18^ using PAL and other clays, and given a primary study about the influence of clays. Qu *et al*. prepared superhydrophobic, superoleophobic/superhydrophilic and superamphiphobic materials from kaolin^34^. Inspired by the famous Maya Blue, we also prepared colorful super anti-wetting coatings using PAL^23,24,35^. Although encouraging results have been obtained, clay-based super anti-wetting coatings are in their infant state. There are a lot of scientific questions remained to be answered in the field. For example, which type of clay or which one is the most suitable for preparation of super anti-wetting coatings? How do the microstructures and physicochemical properties of clays affect the properties and stability of these super anti-wetting surfaces? Can modification of clays improve comprehensive properties of super anti-wetting coatings? The answers to these questions will pave the way for the development of clay-based super anti-wetting surfaces.

Here, we report a comprehensive study about preparation of SAPCs from abundant nanoclays with fibrous, plate-like and porous microstructures. Fifteen kinds of clays, such as PAL, MMT and diatomite, with different microstructures and physicochemical properties were studied. The fibrous clays are superior to the plate-like and porous ones in forming SAPCs. The SAPCs based on fibrous clays feature high superamphiphobicity for various liquids down to a surface tension of 23.8 mN m^−1^ (*n*-decane), and high mechanical, chemical and environmental stability. The superamphiphobicity and stability depend on microscale and nanoscale surface morphology of the coatings, which are controllable by the microstructures of nanoclays and their acid activation.

## Results

### Preparation of Clay*@*fluoroPOS suspensions and SAPCs

**Figure 1:**
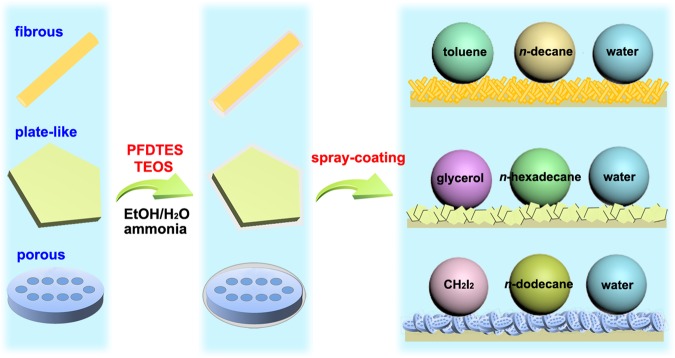
Preparation of the SAPCs. Schematic illustration for preparation of SAPCs from clays with fibrous, plate-like and porous microstructures.

The clay-based SAPCs are prepared by the combination of *1H*, *1H*, *2H*, *2H*-perfluorodecyltriethoxysilane (PFDTES), tetraethoxysilane (TEOS) and clays with various microstructures and physicochemical properties (Fig. 1). By a modified Stöber method, the clays were successfully modified with polymerized perfluoroalkylsilane (fluoroPOS) via hydrolytic condensation of PFDTES and TEOS^36^. Catalyzed by ammonia, hydrolytic condensation of PFDTES and TEOS happened on the surface of clays in the ethanol aqueous solutions by formation of the Si-O-Si bonds between clays and fluoroPOS (Supplementary Fig. S1). In this way, the clay*@*fluoroPOS suspensions were obtained. The clay*@*fluoroPOS SAPCs were readily prepared by spray-coating the suspensions onto glass slides.

The FTIR spectra of three representative clays and the corresponding clay*@*fluoroPOS coatings are shown in Supplementary Fig. S1. In the spectra of PAL, Ca^2+^-MMT and kaolinite, the bands at 3400–3700 cm^−1^ are attributed to stretching vibration of the -OH groups. After modification with PFDTES and TEOS, the absorption bands of the C-F groups appeared at 1210 and 1215 cm^−1^ in the spectra of PAL*@*fluoroPOS, Ca^2+^-MMT*@*fluoroPOS and kaolinite*@*fluoroPOS^37^. The bands at 1032–1036 cm^−1^ are attributed to stretching vibration of the Si-O groups of clays, and the Si-O-Si bonds between clays and fluoroPOS. The bands at 1148–1150 cm^−1^ are attributed to the silsesquioxane of fluoroPOS. In addition, the bands attributed to the -OCH_2_CH_3_ groups of PFDTES and TEOS were not detected. These results indicate complete hydrolysis of silanes and formation of clay*@*fluoroPOS.

### Effect of Clays on superamphiphobicity

Similar to steel reinforced concrete, clays act as the skeleton of the clay*@*fluoroPOS coatings. Thus, the microstructure of clays has a great influence on superamphiphobicity of the coatings. Fifteen kinds of clays were used to prepare the clay*@*fluoroPOS coatings (Supplementary Table S1). *n*-Decane (5 μL) was used as the probe liquid to show the difference in superamphiphobicity among the coatings, as no difference could be seen if water was used. Water drops have very high contact angles (CA_water_ > 160°) and low SA_water_ (<3°) on the surfaces of all the coatings. Table 1 listed the CA_*n*-decane_ and the SA_*n*-decane_ of the clay*@*fluoroPOS coatings.

**Table 1:** Superamphiphobicity of the coatings based on different clays.

Clays	Structure	*C*_clay_/(g L^−1^)	CA_*n*-decane_/°	SA_*n*-decane_/°
PAL	fibrous	10.0	153.6 ± 2.3	14.8 ± 1.8
Halloysite		5.0	155.0 ± 1.7	36.3 ± 1.9
Sepiolite		10.0	149.9 ± 2.3	39.3 ± 1.8
Illite	platelet-like	20.0	153.8 ± 0.3	39.7 ± 1.2
White mica		12.0	149.1 ± 0.7	44.5 ± 3.0
Ca^2+^-MMT		20.0	150.1 ± 1.0	59.8 ± 2.8
Kaolinite		20.0	147.9 ± 0.4	—
Rectorite		20.0	144.5 ± 0.7	—
Li^+^-MMT		10.0	139.8 ± 1.3	—
Laponite RD		20.0	139.6 ± 1.0	—
Na^+^-MMT		15.0	138.3 ± 1.3	—
Vermiculite		×	×	×
Black mica		×	×	×
Hydrotalcite		×	×	×
Diatomite	porous	10.0	150.0 ± 1.2	26.0 ± 1.7

CA_*n*-decane_ and SA_*n*-decane_ of the clay*@*fluoroPOS coatings prepared using different clays. The coatings with *C*_clay_ of 5–20 g L^−1^ were prepared for all the clays, and the coating with the highest superamphiphobicity was listed. “—”means the *n*-decane drops adhered strongly to the coatings even they were turned 180°. “x” means the clay*@*fluoroPOS coating cannot be formed via spray-coating.

**Figure 2:**
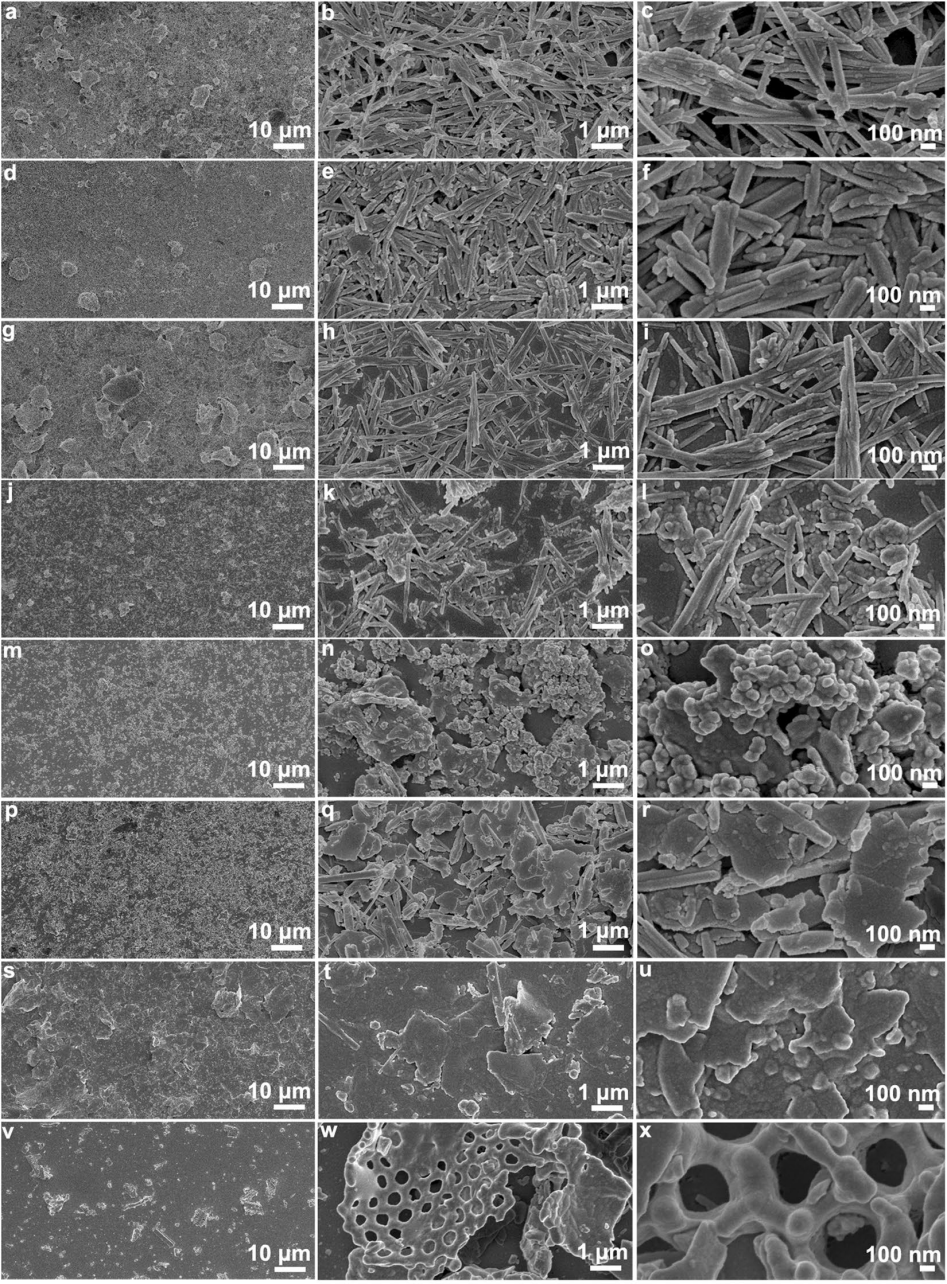
SEM images of different clays. (**a**–**c**) PAL, (**d**–**f**) halloysite, (**g**–**i**) sepiolite, (**j**–**l**) illite, (**m**–**o**) Ca^2+^-MMT, (**p**–**r**) kaolinite, (**s**–**u**) rectorite, and (**v**–**x**) diatomite.

According to their microstructure, the clays in Table 1 can be divided into the following categories: (1) fibrous clays including PAL, sepiolite and halloysite; (2) platelet-like clays including but not limited to MMT, kaolinite, vermiculite, mica and rectorite; (3) porous ones like diatomite. According to the results of extensive experiments, the coatings from fibrous clays show higher superamphiphobicity than the coatings from platelet-like clays. On all the coatings from fibrous clays, the *n*-decane drops are in the Cassie-Baxter state. The superamphiphobicity of the diatomite*@*fluoroPOS coating is higher than all the coatings in Table 1 expect for the PAL*@*fluoroPOS coating. The difference in superamphiphobicity is closely related to surface morphology of the coatings originating from the microstructure of the clays (Fig. 2). The fibrous clays and porous diatomite are helpful in forming coatings with reentrant surface morphology, therefore resulting in coatings with higher superamphiphobicity than those from platelet-like clays (Fig. 1).

For the coatings from fibrous clays, the PAL*@*fluoroPOS coating has a CA_*n*-decane_ of 153.6° and a SA_*n*-decane_ of 14.8°. However, the *n*-decane drops cannot roll off the coatings based on halloysite and sepiolite until the coatings were tilted up to 36.3° and 39.3°, respectively. This is owing to the differences in length and diameter of these fibrous clays. The PAL nanorods are 300–800 nm in length and 20–50 nm in diameter (Fig. 2a–c). Some of the PAL nanorods exist as crystal bundles or aggregates owing to the strong hydrogen bonding and Van der Waals’ interaction among them^27^. Halloysite is in the form of short nanotubes 100–500 nm in length and 50–150 nm in diameter (Fig. 2d–f). Sepiolite nanofibers 1–5 μm in length and 10–50 nm in diameter exist as bundles (Fig. 2g–i). The fibrous clay with moderate aspect ratio results in SAPCs with lower SA_*n*-decane_ by forming better reentrant surface morphology. The aspect ratio of halloysite nanotubes is too small, whereas the sepiolite nanofibers are flexible owing to the very high aspect ratio.

For the coatings from platelet-like clays, only the coatings based on illite, Ca^2+^-MMT and white mica have the CA_*n*-decane_ around 150°, and the *n*-decane drops can roll off the coatings but the SA_*n*-decane_ are very high (40–60°). The CA_*n*-decane_ of the coatings from kaolinite, rectorite, Li^+^-MMT, Laponite RD and Na^+^-MMT are lower, and the *n*-decane drops adhere stably to the coatings even the coatings were turned 180°. This means the *n*-decane drops on the surface of the coatings are in the Wenzel state. The difference in the superamphiphobicity is owing to the diversity in microstructure of the platelet-like clays. Illite contains ribbon-like platelets with varied aspect ratio and nanoparticles 20–50 nm in size (Fig. 2j–l). The morphology of illite is more close to the fibrous clays than the others. Kaolinite is composed of platelets 0.3–1.2 μm in size (Fig. 2m–o). The morphology of rectorite is very similar to that of kaolinite, but the platelets are bigger (Fig. 2p–r). Na^+^-MMT is composed of slightly curly platelets stacking together (Fig. 2s–u). In the case of vermiculite, black mica and hydrotalcite, the dispersion stability of the clay*@*fluoroPOS suspensions is very low, which makes it impossible to prepare the corresponding coatings via spray-coating.

### Effects of clay content and acid activation on superamphiphobicity

Acid activation is commonly employed to improve physicochemical properties of clays and remove soluble impurities^38,39^. On the basis of the above results, eleven kinds of clays were activated using 2 M HCl_(aq)_. The influences of clay content and acid activation on superamphiphobicity and surface morphology of the coatings were studied.

**Figure 3:**
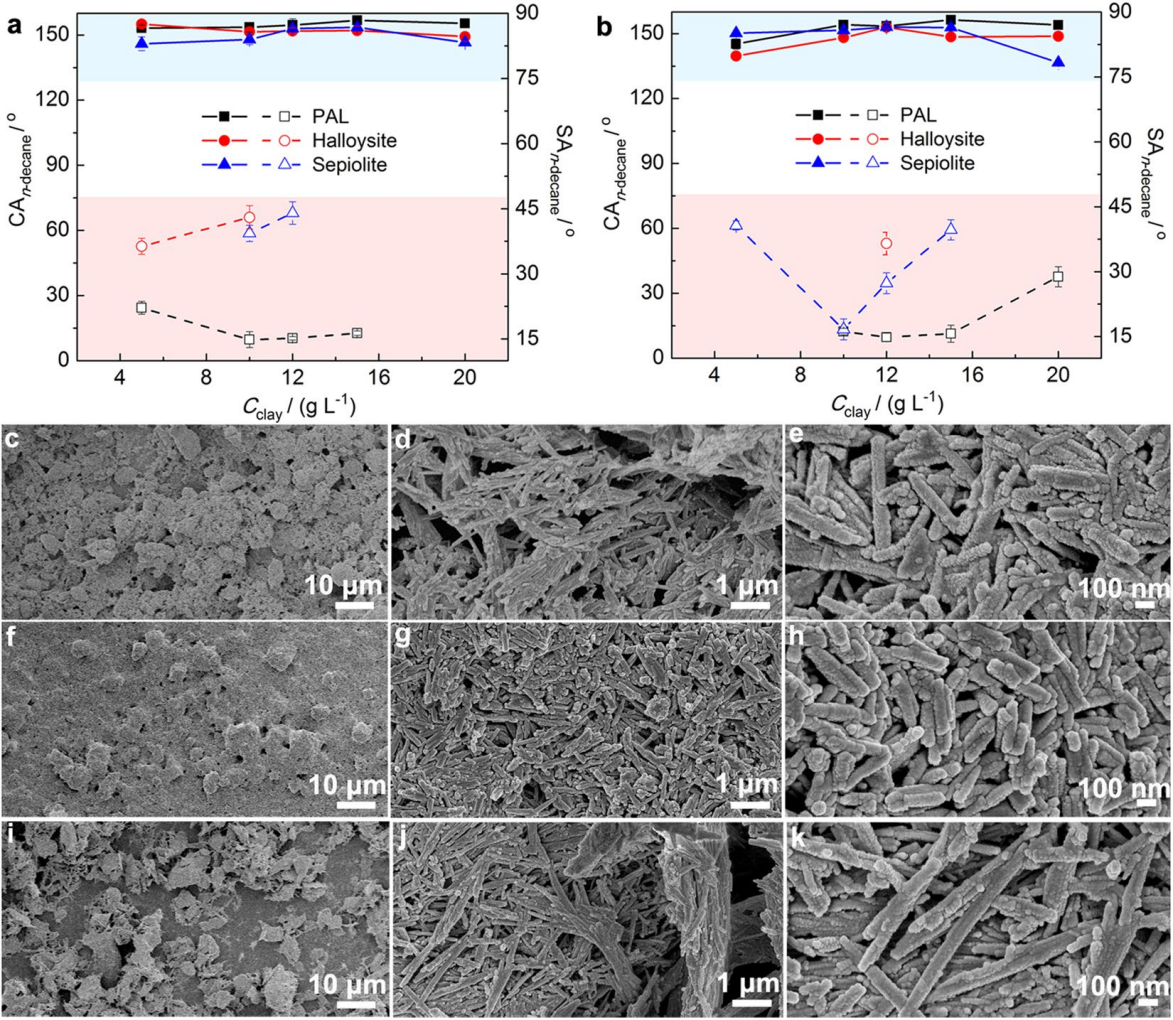
Superamphiphobicity and microstructure of the coatings based on fibrous clays. CA_*n*-decane_ and SA_*n*-decane_ of the clay*@*fluoroPOS coatings from (**a**) pristine and (**b**) acid activated fibrous clays. SEM images of the coatings from acid activated (**c**–**e**) PAL (*C*_PAL_ = 12 g L^−1^), (**f**–**h**) halloysite (*C*_halloysite_ = 12 g L^−1^) and (**i**–**k**) sepiolite (*C*_sepiolite_ = 10 g L^−1^). The solid lines represent the CA_*n*-decane_, and the dash lines represent the SA_*n*-decane_.

Figure 3a shows superamphiphobicity of the coatings prepared using the pristine fibrous clays. For the PAL*@*fluoroPOS coatings, the CA_*n*-decane_ remained in the range 153.2°–156.8° with increasing the *C*_PAL_ from 5 to 15 g L^−1^, and meanwhile the SA_*n*-decane_ reduced from 22.2° to 14.8°–16.3°. With increasing the *C*_PAL_ to 20 g L^−1^, the *n*-decane drops became sticky on the coating although there was no obvious change in the CA_*n*-decane_, implying transition of the droplets to the Wenzel state. The difference in the CA_*n*-decane_ among the coatings from PAL, halloysite and sepiolite is small when the *C*_clay_ is 5–20 g L^−1^. However, the SA_*n*-decane_ has big difference. The SA_*n*-decane_ of the coatings from halloysite and sepiolite are higher than those of the PAL*@*fluoroPOS coatings. Also, the *n*-decane drops are in the Cassie-Baxter state in a wider range of *C*_clay_ in the case of PAL. In general, the coatings from the pristine fibrous clays have the optimal superamphiphobicity when the *C*_clay_ is ca. 10 g L^−1^.

Acid activation of the fibrous clays resulted in a little bigger change of the CA_*n*-decane_ with increasing the *C*_clay_, and also larger difference in the CA_*n*-decane_ among the coatings from PAL, halloysite and sepiolite (Fig. 3b). However, the change in the SA_*n*-decane_ was evident after acid activation. For the coatings from activated PAL, the *n*-decane drops are sticky on the coatings when the *C*_PAL_ is below 10 g L^−1^. Activation of PAL has no influence on the SA_*n*-decane_ when the *C*_PAL_ is 10–15 g L^−1^. The *n*-decane drops still could roll off the coating with further increasing the *C*_PAL_ to 20 g L^−1^ in spite of increase in the SA_*n*-decane_. Activation of halloysite does not reduce the SA_*n*-decane_, and a higher *C*_halloysite_ is necessary to keep the *n*-decane drops in the state of Cassie-Baxter. Differently, activation of sepiolite is helpful to reduce the SA_*n*-decane_. In a wide range of *C*_sepiolite_ (5–15 g L^−1^), the *n*-decane drops can roll down from the coatings. Similar to the PAL*@*fluoroPOS coating, the coating with 10 g L^−1^ *C*_sepiolite_ has a SA_*n*-decane_ of 16.7°.

The difference in superamphiphobicity of the coatings from the fibrous clays is closely related to their surface morphology. The micrographs of the coatings with the lowest SA_*n*-decane_ from the activated fibrous clays are shown in Fig. 3c–k. All the three coatings have hierarchical micro-/nanostructures, which are built by the fluoroPOS-modified clay nanofibers with fluoroPOS as the crosslinker. The fluoroPOS-modified clay nanofibers form the nanoscale roughness, whereas their aggregates form the microscale roughness. The hierachical micro-/nanostructures are helpful to stably trap some air at the interface of the coatings and the liquids, which leads to rolling of the *n*-decane drops off the coatings. At low magnification, the surface morphology of the coatings is obviously different from each other (Fig. 3c,f,i). The microscale roughness of the coating from halloysite is lower than that from PAL or sepiolite. In addition, the coating from PAL is even-textured compared to that from sepiolite (Fig. 3c,i), which is owing to serious aggregation of the sepiolite nanofibers induced by fluoroPOS. On the other hand, at high magnification, the surface morphology of the coatings is very similar to each other (Fig. 3e,h,k). Thus, the difference in the microscale surface morphology should be responsible for the difference in superamphiphobicity.

**Figure 4:**
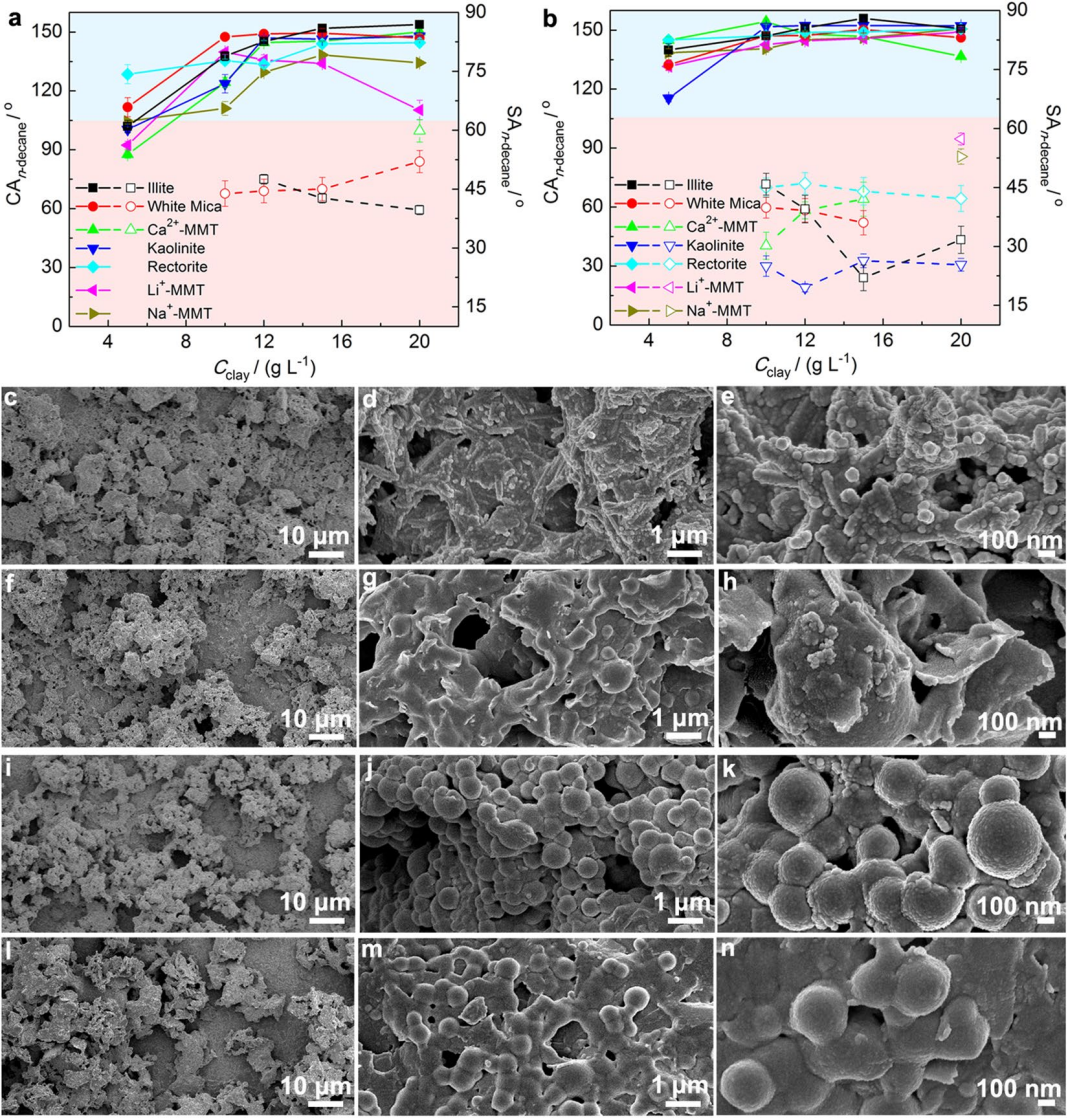
Superamphiphobicity and microstructure of the coatings based on platelet-like clays. CA_*n*-decane_ and SA_*n*-decane_ of the coatings from (**a**) pristine and (**b**) acid activated platelet-like clays. SEM images of the coatings from acid activated (**c**–**e**) illite, (**f**–**h**) Ca^2+^-MMT, (**i**–**k**) kaolinite, and (**l**–**n**) rectorite. The solid lines represent the CA_*n*-decane_, and the dash lines represent the SA_*n*-decane_.

The superamphiphobicity of the coatings from the pristine platelet-like clays is shown in Fig. 4a. The CA_*n*-decane_ increased with increasing the *C*_clay_ from 5 to 20 g L^−1^ for all the coatings expect for that from Li^+^-MMT. For the Li^+^-MMT*@*fluoroPOS coatings, a decrease in the CA_*n*-decane_ was observed when the *C*_Li+-MMT_ was over 10 g L^−1^. On the other hand, the *n*-decane drops could only roll off the coatings from illite, white mica and Ca^2+^-MMT when their concentrations were in proper ranges, e.g., 12–20 g L^−1^ of illite, 10–20 g L^−1^ of white mica and 20 g L^−1^ of Ca^2+^-MMT. For the other pristine platelet-like clays, the *n*-decane drops adhered stably to the clay*@*fluoroPOS coatings when the *C*_clay_ was in the range 5–20 g L^−1^_._

Acid activation of the platelet-like clays resulted in obvious improvement in superamphiphobicity of the coatings as shown in Fig. 4b. When the *C*_clay_ was in proper ranges, the CA_*n*-decane_ became higher, and the droplets can roll down from all the coatings. With increasing the *C*_clay_ of the activated platelet-like clays, there are two different trends in the SA_*n*-decane_ and CA_*n*-decane_. For rectorite, Na^+^-MMT and Li^+^-MMT, when the *C*_clay_ increased from 5 to 20 g L^−1^, the SA_*n*-decane_ decreased and the CA_*n*-decane_ increased. For Ca^2+^-MMT, illite, kaolinite and white mica, when the *C*_clay_ increased to 15 g L^−1^, the CA_*n*-decane_ increased, and the SA_*n*-decane_ decreased. Then, the SA_*n*-decane_ increased and the CA_*n*-decane_ decreased when the *C*_clay_ further increased to 20 g L^−1^.

The micrographs of the coatings with the lowest SA_*n*-decane_ from the four activated platelet-like clays are shown in Fig. 4c–n. All the four coatings showed high micro-/nanoscale roughness, which is responsible for the high superamphiphobicity. At low magnification, the surface morphology of the coatings is very similar to each other (Fig. 4c,f,i and l). There are a lot of aggregates of similar size on the surface of the coatings. However, at high magnification, the surface morphology of the coatings is completely different from each other (Fig. 4d,g,j and m). The nanoscale roughness of the coating from kaolinite is higher than that from rectorite, illite or Ca^2+^-MMT. In addition, the coating from kaolinite has many small protrusions which are responsible for the lower SA_*n*-decane_ (Fig. 4j and m). So, the difference in the nanoscale surface morphology should be responsible for the difference in superamphiphobicity.

**Figure 5:**
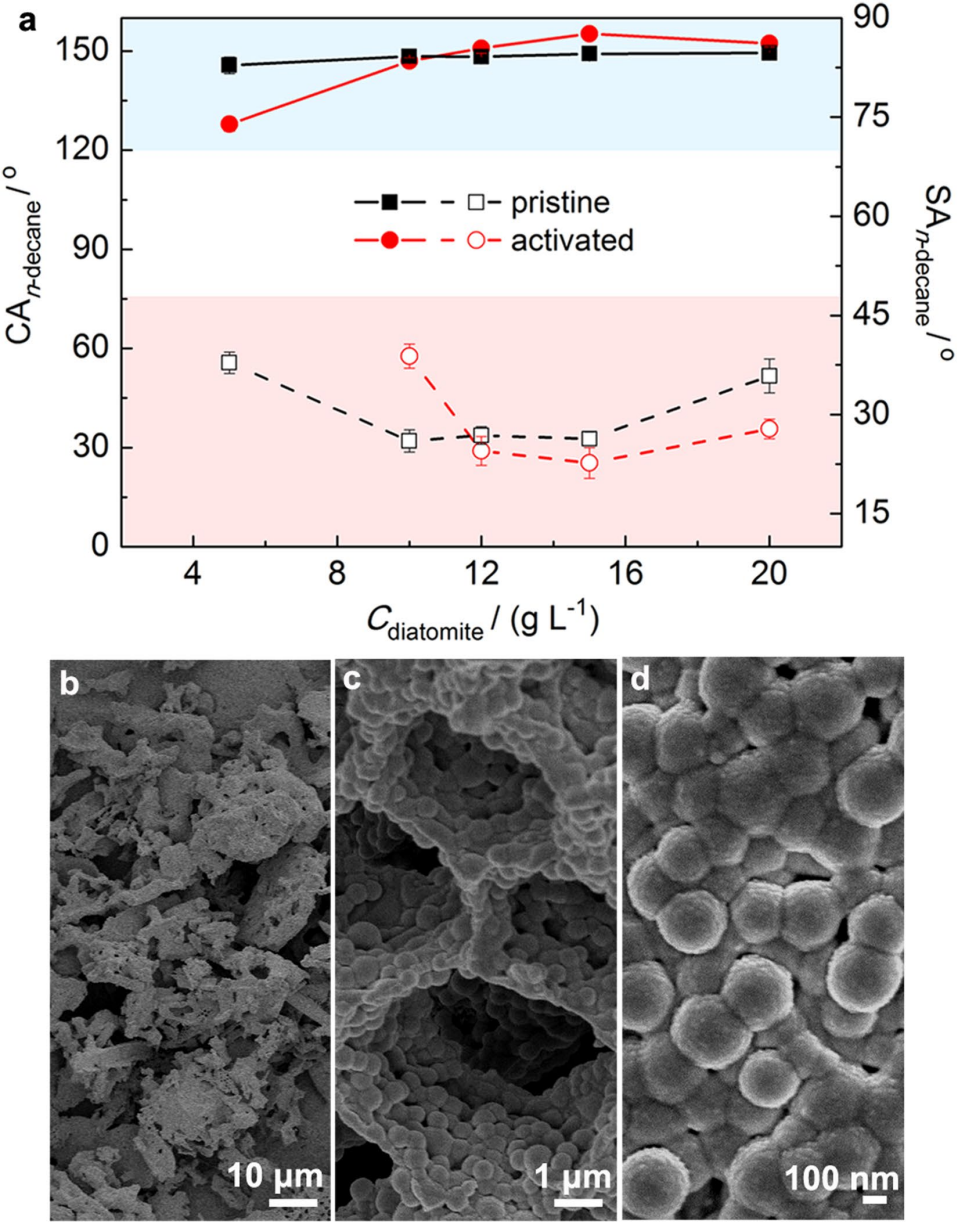
Superamphiphobicity and microstructure of the coatings based on diatomite. CA_*n*-decane_ and SA_*n*-decane_ of the coatings from (**a**) pristine and acid activated diatomite, and (**b**–**d**) SEM images of the coating from acid activated diatomite (*C*_diatomite_ = 15 g L^−1^). The solid lines represent the CA_*n*-decane_, and the dash lines represent the SA_*n*-decane_.

The superamphiphobicity of the coatings from the pristine and activated diatomite is shown in Fig. 5a. For the coatings from pristine diatomite, the CA_*n*-decane_ was in the range 145.6°–149.3° with increasing the *C*_diatomite_ from 5 to 20 g L^−1^. Meanwhile, the SA_*n*-decane_ reduced from 37.8° to 26.0° with increasing the *C*_diatomite_ to 10 g L^−1^. Increasing the *C*_diatomite_ to 20 g L^−1^ caused increase in the SA_*n*-decane_ to 35.8°.

Acid activation of diatomite affected the CA_*n*-decane_ and SA_*n*-decane_, and slightly improved the superamphiphobicity. The CA_*n*-decane_ increased from 127.8° to 155.2° with increasing the *C*_diatomite_ from 5 to 15 g L^−1^. Although the *n*-decane drops were sticky on the coatings when the *C*_diatomite_ was below 10 g L^−1^, the SA_*n*-decane_ reduced to 22.6° with increasing the *C*_diatomite_ from 10 to 15 g L^−1^. Then, the SA_*n*-decane_ changed to 27.8° with further increasing the *C*_diatomite_ to 20 g L^−1^.

The SEM images of the coating with the lowest SA_*n*-decane_ from the activated diatomite are shown in Fig. 5b–d. The coating is rough in both the microscale and the nanoscale. The microscale roughness is owing to aggregates of the activated diatomite induced by fluoroPOS (Fig. 5b). There are many micropores on the surface of the coating because diatomite is porous (Fig. 5c). Also, there are many nanoparticles 200–300 nm in diameter on the wall of the micropores (Fig. 5d). The high superamphiphobicity of the diatomite*@*fluoroPOS coatings relies on the hierachical micro-/nanostructures.

Besides superamphiphobicity and the surface morphology, acid activation of the clays also enhanced stability of the clay*@*fluoroPOS suspensions. This is helpful to form uniform coatings via spray-coating. The enhanced stability of the clay*@*fluoroPOS suspensions is attributed to the fact that the zeta potentials of activated clays became more negative than pristine ones (Supplementary Table S2). Acid activation could break the Si-O-Si bonds and remove the impurities of clays, which produced more negatively charged sites^40,41^. Zeta potential is commonly used as an index of the stability of suspensions^42^.

After understanding the effects of clays and their acid acidification on superamphiphobicity and micro-/nanostructures of the clay*@*fluoroPOS coatings, superamphiphobicity of the coatings based on acid activated clays was studied in detail. Three representative coatings based on activated PAL, kaolinite and Ca^2+^-MMT were presented in Table 2.

**Table 2:** Superamphiphobicity.

Liquids	PAL*@*fluoroPOS		Kaolinite*@*fluoroPOS		Ca^2+^-MMT*@*fluoroPOS		Surface tension (mN m^−1^, 20 °C)
	CA**/**°	SA**/**°	CA**/**°	SA**/**°	CA**/**°	SA**/**°	
Water	163.7 ± 1.5	2.5 ± 0.5	160.8 ± 0.2	4.5 ± 0.5	160.4 ± 0.8	5.5 ± 0.5	72.8
Glycerol	161.7 ± 0.9	3.5 ± 0.5	159.8 ± 0.4	5.5 ± 0.5	157.6 ± 0.4	6.7 ± 0.5	64.0
Diiodomethane	158.6 ± 0.5	4.8 ± 0.7	158.0 ± 1.1	8.2 ± 0.7	155.5 ± 0.6	11.7 ± 0.8	50.8
*N*-Methyl-2-pyrrolidone	157.1 ± 0.2	6.7 ± 0.5	157.5 ± 0.6	12.2 ± 1.2	155.3 ± 0.5	14.8 ± 1.2	40.8
Toluene	157.5 ± 0.7	8.2 ± 0.8	155.4 ± 0.7	15.0 ± 1.4	153.5 ± 0.6	21.5 ± 2.2	28.4
*n*-Hexadecane	157.1 ± 0.8	8.3 ± 0.7	155.6 ± 0.8	15.3 ± 1.2	153.3 ± 0.4	23.5 ± 1.6	27.5
*n*-Dodecane	155.6 ± 1.1	11.0 ± 0.9	153.9 ± 0.9	17.7 ± 1.0	152.5 ± 0.9	27.3 ± 0.5	25.4
*n*-Decane	154.7 ± 0.9	14.8 ± 0.8	153.2 ± 0.7	20.2 ± 1.2	152.3 ± 0.8	29.3 ± 1.4	23.8

CAs and SAs of diverse liquids on the PAL*@*fluoroPOS, kaolinite*@*fluoroPOS and Ca^2+^-MMT*@*fluoroPOS coatings from acid activated clays at 25 °C.

**Figure 6:**
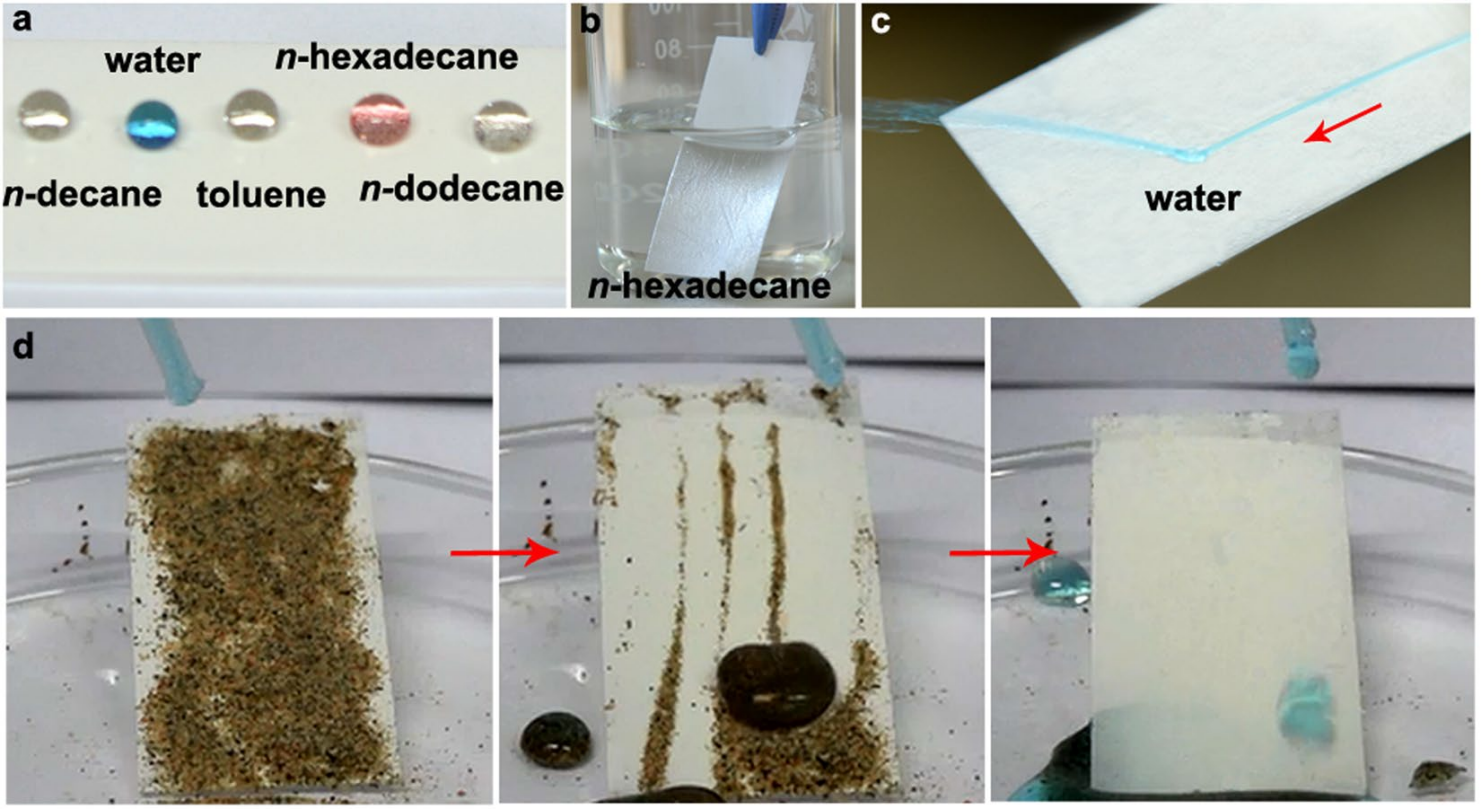
Superamphiphobicity of the PAL*@*fluoroPOS coating. Photographs of the coatings (**a**) with different liquids, (**b**) in *n*-hexadecane, (**c**) with a stream of water flowing away and (**d**) showing self-cleaning property.

For the PAL*@*fluoroPOS coating, all the tested liquids have CAs above 154° (Fig. 6a) and SAs below 15°. The drops can roll down from all the tilted coating, which means extremely weak interaction at the solid-liquid interface. Beyond that, the high superamphiphobicity of the coating was also demonstrated by the following facts. Once immersed in *n*-hexadecane, a silver mirror-like phonomenon was observed on the surface of the coating, and the coating was entirely dry after taken out of *n*-hexadecane (Fig. 6b). A stream of water can flow away the coating easily (Fig. 6c). The superamphiphobicity of the coatings are in the order of PAL*@*fluoroPOS > kaolinite*@*fluoroPOS > Ca^2+^-MMT*@*fluoroPOS regarding their CAs and SAs for all the liquids in Table 2. Moreover, all the three coatings show self-cleaning property (Fig. 6d). The dirt on the coatings can be easily removed by the liquids in Table 2.

The stability of the representative PAL*@*fluoroPOS, kaolinite*@*fluoroPOS and Ca^2+^-MMT*@*fluoroPOS coatings from acid activated clays was evaluated. The stability was studied via water jetting, dipping in corrosive liquids and so on. The CA_*n*-decane_ and SA_*n*-decane_ were recorded after these tests to evaluate the changes in superamphiphobicity. It should be noted that the changes in the SAs, especially for the liquids of low surface tension, are strongly suggested to be shown after stability tests of super anti-wetting coatings. Without the SAs, the CAs themselves are not sufficient to show stability of such coatings.

**Figure 7:**
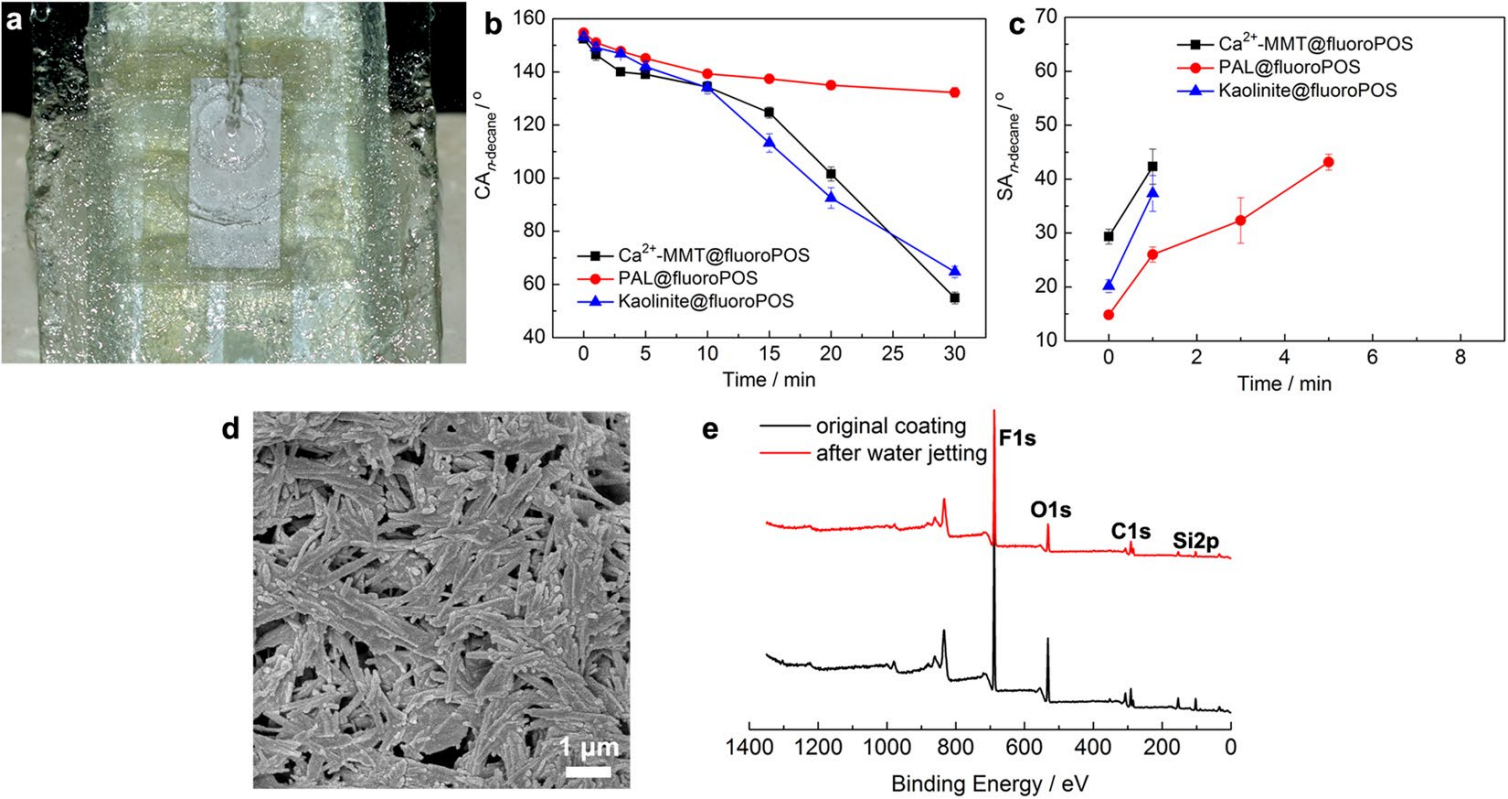
Mechanical stability of the PAL*@*fluoroPOS coating. (**a**) Photograph of water jetting at 50 kPa, (**b**) CA_*n*-decane_ and (**c**) SA_*n*-decane_ of the three representative coatings from acid activated clays in the water jetting test, (**d**) SEM image and (**e**) XPS spectrum of the PAL*@*fluoroPOS coating after 30 min water jetting.

The mechanical stability of the clay*@*fluoroPOS coatings was studied by the intensive water jetting test^18,43^. Water jet at 50 kPa impacted the 45° inclined coatings for half an hour (Fig. 7a and Supplementary Video S1). When the water jetting time increased, the SA_*n*-decane_ increased and the CA_*n*-decane_ decreased (Fig. 7b,c). For the PAL*@*fluoroPOS coating, the CA_*n*-decane_ was 150.9° and the SA_*n*-decane_ was 26° after water jetting for 1 min. The CA_*n*-decane_ was 139.2°, but the *n*-decane drops adhered to the coating after 10 min, indicating transition of the *n*-decane drops from the Cassie-Baxter state to the Wenzel state. It should be noted that the coating was still perfectly superhydrophobic after water jetting for 30 min, and no change in the CA_water_ and SA_water_ was detected. The surface micrograph and the surface chemical composition of the coatings have been studied after 30 min water jetting. There is no big change in the surface morphology as shown in Fig. 7d. In addition, according to the X-ray photoelectron spectroscopy (XPS), the F 1 s peak is as strong as before (Fig. 7e) and the F content is 45.05 at.%, similar to the new coating (Supplementary Table S3). Mechanical stability of the coatings is in the order of PAL*@*fluoroPOS > kaolinite*@*fluoroPOS ≈ Ca^2+^-MMT*@*fluoroPOS regarding the changes in the CA_*n*-decane_ and the SA_*n*-decane_.

The chemical stability of the representative clay*@*fluoroPOS coatings were studied by UV irradiation or immersion in various corrosive liquids (Supplementary Table S4). The PAL*@*fluoroPOS coating was quite stable after UV irradiation for 24 h, however, the other two coatings showed slight decline in superamphiphobicity. For example, after UV irradiation for 24 h, the SA_*n*-decane_ of the kaolinite*@*fluoroPOS coatings increased from 20.2° to 27.3°. After immersed in different corrosive solutions (e.g., 1 M HCl_(aq)_ and ethanol) for 24 h, there was no evident change in the CA_*n*-decane_ and the SA_*n*-decane_ of the PAL*@*fluoroPOS coating, except for saturated NaOH_(aq)_ and 98% H_2_SO_4_. The CA_*n*-decane_ decreased to 151.5° and the SA_*n*-decane_ increased to 30.1° after immersed in saturated NaOH_(aq)_ for 24 h. The *n*-decane drops adhered to the coating after immersed in 98% H_2_SO_4_ for 24 h. After immersed in the aforementioned corrosive liquids for 24 h, the kaolinite*@*fluoroPOS and Ca^2+^-MMT*@*fluoroPOS coatings showed very similar changes in the SA_*n*-decane_ and the CA_*n*-decane_. The chemical stability of the coatings is in the order of PAL*@*fluoroPOS > kaolinite*@*fluoroPOS > Ca^2+^-MMT*@*fluoroPOS based on the data in Supplementary Table S4.

**Figure 8:**
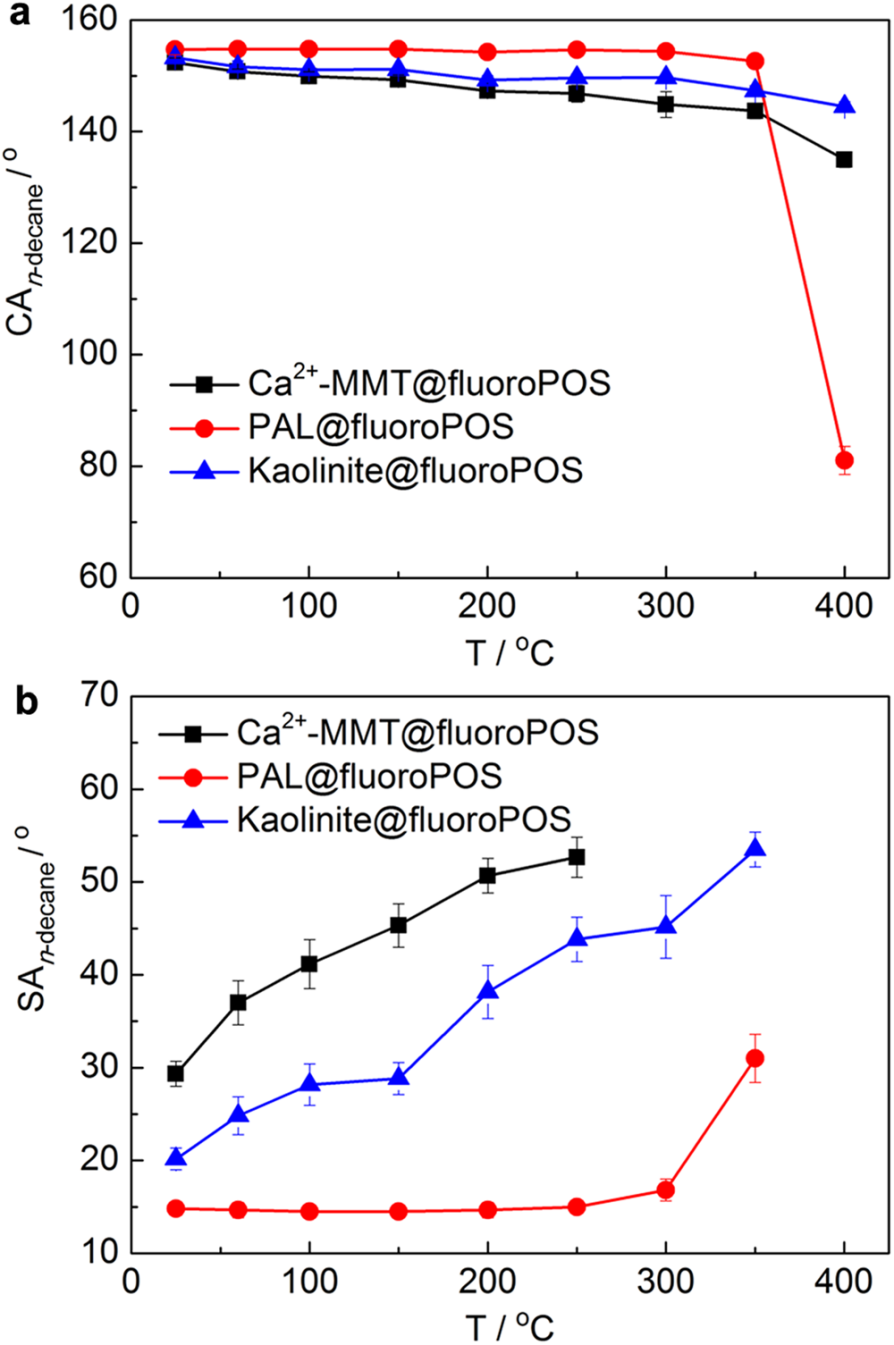
Thermal stability of the PAL*@*fluoroPOS coating. (**a**) CA_*n*-decane_ and (**b**) SA_*n*-decane_ of the three clay*@*fluoroPOS coatings fabricated using acid activated clays after kept at different temperature in air for 1 h.

The thermal stability of the representative clay*@*fluoroPOS coatings is shown in Fig. 8. After being kept for one hour at temperature up to 300 °C, the PAL*@*fluoroPOS coating remained superamphiphobic (CA_*n*-decane_ = 154.3°, SA_*n*-decane_ = 16.8°) and the *n*-decane drops can roll down from the coating. The SA_*n*-decane_ increased to 31° at 350 °C, then the *n*-decane drops adhered stably to the coating (CA_*n*-decane_ = 81.1°) at 400 °C. This is because of thermal oxidation of fluoroPOS in the coating. The kaolinite*@*fluoroPOS and Ca^2+^-MMT*@*fluoroPOS coatings show similar change in the CA_*n*-decane_, and the difference among the coatings is small with increasing the temperature, except for 400 °C. However, the difference in the SA_*n*-decane_ among the coatings is quite evident. For the PAL*@*fluoroPOS coating, the SA_*n*-decane_ did not show obvious change with increasing the temperature up to 300 °C. However, the SA_*n*-decane_ increased gradually to ca. 45° at 300 °C for the kaolinite*@*fluoroPOS coating, and to above 50° at 250 °C for the Ca^2+^-MMT*@*fluoroPOS coating.

## Discussion

In summary, we have carried out a comprehensive study about preparation of SAPCs from abundant nanoclays with diverse microstructures such as fibrous, plate-like and porous. Compared to the plate-like and porous clays, the fibrous nanoclays with moderate aspect ratio, e.g., palygorskite, are the most suitable for preparation of SAPCs by forming the optimal reentrant surface morphology. Acid activation of clays could evidently enhance superamphiphobicity of the coatings. The superamphiphobicity relies on microscale and nanoscale surface morphology of the coatings, which are determined by the microstructure and content of the nanoclays. The coatings based on acid activated PAL show high superamphiphobicity for various liquids with low surface tension. Meanwhile, the coatings have excellent mechanical stability against intense water jetting, high chemical stability after UV irradiation and immersion in various corrosive liquids, and high thermal stability up to 350 °C. The SAPCs from abundant nanoclays will find applications in anti-adhesion and anti-climbing of liquids of low surface tension such as oils, organic solvents, polymer solution and surfactant solutions, because of high superamphiphobicity and stability of the coatings. The findings in this study will promote the progress of SAPCs, and pave the way for the development of clay-based super anti-wetting coatings.

## Methods

### Materials

PAL was purchased from Jiangsu, China. Kaolinite and vermiculite were purchased from Fujian, China. Illite and rectorite were purchased from Hubei, China. Mica was purchased from Hebei, China. Na^+^-MMT was purchased from Shandong, China. Ca^2+^-MMT and Li^+^-MMT was purchased from Henan, China. Halloysite, diatomite, hydrotalcite and sepiolite were purchased from Sigma-Aldrich. Laponite RD was supplied by Southern Clay Products, Inc. For acid activation, the clays (15 g) were suspended in the 2 M HCl aqueous solutions (150 mL) at room temperature and magnetically stirred for 2 h. The acid activated clays were washed with deionized water until pH 6, and then dried at 105 °C to a constant weight. The clays were sifted using a 200 mesh sieve. Glass slides (24 × 50 mm) were purchased from Menzel, Germany. PFDTES (97%) and TEOS (99.9%) were bought from Gelest. Ethanol, ammonia, CH_2_I_2_, toluene *n*-dodecane, *n*-hexadecane and *n*-decane were bought from China National Medicines Co. Ltd.

### Preparation of Clay*@*fluoroPOS coatings

Ethanol (44 mL) was mixed with 6 mL of an aqueous ammonia solution, and then 0.25–1.0 g of clay was added. After 30 min ultrasonication, PFDTES (22.7 mM) and TEOS (8.9 mM) were added into the suspensions. Then, the suspensions were stirred vigorously for 6 h at room conditions. As depicted in Fig. 1, by means of spraying the suspensions (6 mL) onto glass slides employing an airbrush with 0.2 MPa N_2_, the clay*@*fluoroPOS coatings were fabricated.

## Electronic supplementary material

Supplementary Information

Supplementary video S1
