# Supplementary material for: Biomimetic Super Anti-Wetting Coatings from Natural Materials: Superamphiphobic Coatings Based on Nanoclays
Source: Sci Rep. 2018 Aug 13;8:12062. doi: 10.1038/s41598-018-30586-4 (PMC6089923; doi:10.1038/s41598-018-30586-4)
Supplement: Supplementary file 1 — Supplementary Information [file 41598_2018_30586_MOESM1_ESM.pdf]

Supporting information

Biomimetic Super Anti-Wetting Coatings from Natural Materials: Superamphiphobic Coatings Based on Nanoclays

Jie Dong[1,2] & Junping Zhang[1*]

[1] Key Laboratory of Clay Mineral Applied Research of Gansu Province, and State Key Laboratory of Solid Lubrication, Lanzhou Institute of Chemical Physics, Chinese Academy of Sciences, 730000, Lanzhou, P.R. China

[2] University of Chinese Academy of Sciences, 100049, Beijing, P.R. China

*Correspondence to jpzhang@licp.cas.cn

Characterization. Measurements of CAs, SAs were performed with a Contact Angle System OCA20 (Dataphysics, Germany) equipped with a tilting table. The syringe was positioned in a way that the liquid drops (5 μL) could contact surface of the samples before leaving the needle. Tilting angle of the table was adjustable (0-70°) and allowed the subsequent measurement of the SAs at the same position on the sample. A minimum of six readings were recorded for each sample, and the average values with standard errors were reported. The micrographs of the samples were taken using a field emission SEM (JSM-6701F, JEOL). Before SEM observation, all samples were fixed on aluminum stubs and coated with gold (~7 nm). The FTIR spectra of samples were collected on a Thermo Nicolet NEXUS TM spectrophotometer (Thermo, Madison, USA) in the range of 4000-400 cm-1 using KBr pellets. The XPS spectra of samples were obtained using a VG ESCALAB 250 Xi spectrometer equipped with a Monochromated Al Kα X-ray radiation source and a hemispherical electron analyzer. The spectra were recorded in the constant pass energy mode with a value of 100 eV, and all binding energies were calibrated using the C1s peak at 284.6 eV as the reference.

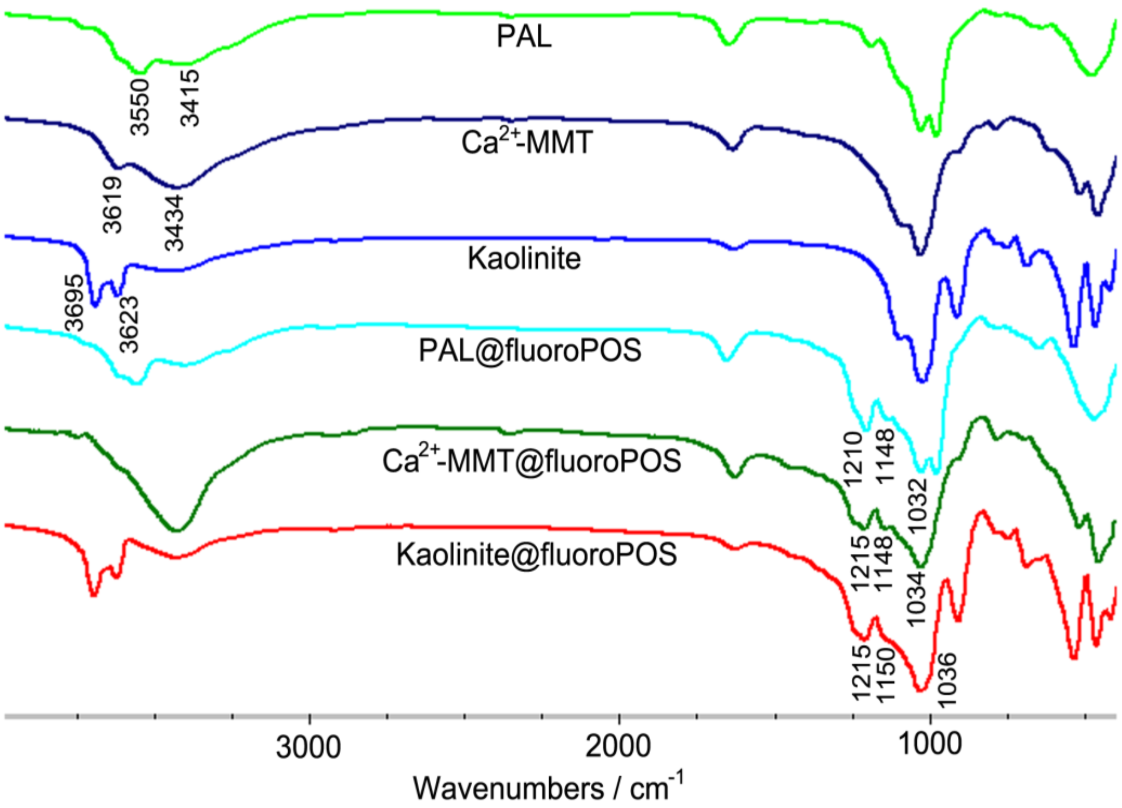

Supplementary Fig. S1. FTIR spectra of PAL, Ca^{2+}-MMT, kaolinite, PAL@fluoroPOS, Ca^{2+}-MMT@fluoroPOS and kaolinite@fluoroPOS.

Clays	Structure	Chemical formula
PAL	fibrous	$Mg_5Si_8O_{20}(OH)_2(OH)_4 \cdot 4H_2O$
Halloysite		$Al_2Si_2O_5(OH)_4 \cdot 2H_2O$
Sepiolite		$Mg_8(H_2O)_4[Si_6O_{16}]_2(OH)_4 \cdot 8H_2O$
Illite	platelet-like	$KAl_2[(OH)_2AlSi_3O_{10}]$
White mica		$KAl_2[AlSi_3O_{10}](OH)_2$
Ca^{2+}-MMT		$(Ca_{0.46}Mg_{0.04}Fe_{0.06})(Al_{1.76}Fe_{0.10}Ti_{0.14})[Al_{0.90}Si_{3.10}O_{10}](OH)_2 \cdot nH_2O$
Kaolinite		$Al_4[Si_4O_{10}](OH)_8$
Rectorite		—
Li^{+}-MMT		$Na_{0.67}(Mg, Li)_6Si_8O_{20}(OH, F)_4$
Laponite RD		$[(Si_8(Mg_{5.34}Li_{0.66})O_{20}(OH)_4)]Na_{0.66}$
Na^{+}-MMT		$(1/2Ca, Na)_{0.7}(Al, Mg, Fe)_4(Si, Al)_8O_{20}(OH)_4$
Vermiculite		$(Mg, Fe, Al)_3[(Si, Al)_4O_{10}](OH)_2 \cdot 4H_2O$
Black mica		$K(Mg, Fe)_3(Si_3Al)O_{10}(OH)_2$
Hydrotalcite		$[Mg_6Al_2(OH)_{16}CO_3] \cdot 4H_2O$
Diatomite	porous	$SiO_2 \cdot nH_2O$

Supplementary Table S1. Structure and chemical formula of different clays.

Clays	Structure	Zeta potentials / mV	
		pristine	acid activated
PAL	fibrous	-17.03	-18.42
Halloysite		-17.34	-23.93
Sepiolite		-16.54	-17.73
Illite	platelet-like	-13.06	-34.74
White Mica		-25.01	-34.74
Kaolinite		-26.91	-34.70
Ca^{2+}-MMT		-30.86	-35.71
Li^{+}-MMT		-31.22	-40.04
Na^{+}-MMT		-32.02	-38.57
Na^{+}-rectorite		-37.05	-38.16
Diatomite	porous	-28.35	-37.23

Supplementary Table S2. Zeta potentials of eleven kinds of clays before and after acid activation.

Coatings	F content / at.%	C/O/F atomic ratio
Original	47.82	1:0.62:1.82
After water jetting	45.05	1:0.53:1.54

Supplementary Table S3. F content and C/O/F atomic ratio on the surface of the PAL@fluoroPOS coating before and after water jetting at 50 kPa for 30 min.

	PAL@fluoroPOS		Kaolinite@fluoroPOS		Ca^{2+}-MMT@fluoroPOS	
	$CA_{n\text{-decane}}$ / °	$SA_{n\text{-decane}}$ / °	$CA_{n\text{-decane}}$ / °	$SA_{n\text{-decane}}$ / °	$CA_{n\text{-decane}}$ / °	$SA_{n\text{-decane}}$ / °
Original coating	154.7 ± 0.9	14.8 ± 0.8	153.2 ± 0.7	20.2 ± 1.2	152.3 ± 0.8	29.3 ± 1.4
UV irradiation	153.6 ± 1.5	14.8 ± 0.8	150.2 ± 2.0	27.3 ± 1.6	149.9 ± 2.9	33.8 ± 2.7
Distilled water, 1 h	153.8 ± 1.7	15.3 ± 0.8	150.9 ± 1.6	20.2 ± 1.2	150.1 ± 0.3	34.7 ± 2.4
Distilled water, 24 h	152.5 ± 1.7	15.8 ± 0.7	146.9 ± 1.4	25.0 ± 1.7	144.0 ± 1.6	36.5 ± 2.7
Ethanol, 1 h	153.8 ± 1.1	15.2 ± 1.0	151.3 ± 1.3	24.3 ± 1.2	150.1 ± 0.3	34.7 ± 2.4
Ethanol, 24 h	151.4 ± 0.7	18.8 ± 0.7	144.3 ± 1.7	28.3 ± 1.8	144.0 ± 1.6	36.5 ± 2.7
1 M $NaOH_{(aq)}$, 1 h	153.8 ± 0.6	14.8 ± 0.8	150.9 ± 1.3	25.7 ± 2.3	150.8 ± 1.3	29.2 ± 1.5
1 M $NaOH_{(aq)}$, 24 h	153.3 ± 0.8	17.8 ± 1.4	140.7 ± 0.6	34.5 ± 3.0	143.9 ± 1.4	34.5 ± 2.6
1 M $HCl_{(aq)}$, 1 h	153.7 ± 1.1	17.0 ± 1.4	151.3 ± 0.5	24.8 ± 1.2	147.7 ± 0.8	30.7 ± 2.1
1 M $HCl_{(aq)}$, 24 h	152.1 ± 1.0	18.5 ± 0.8	147.3 ± 0.9	29.5 ± 2.0	145.5 ± 0.7	34.8 ± 1.2
Saturated $NaCl_{(aq)}$, 1 h	153.6 ± 1.3	16.0 ± 1.2	152.2 ± 0.6	23.8 ± 1.5	150.7 ± 0.7	33.3 ± 2.6
Saturated $NaCl_{(aq)}$, 24 h	152.7 ± 1.8	16.3 ± 1.2	147.3 ± 1.3	35.8 ± 2.3	145.5 ± 1.4	38.7 ± 2.4
Saturated $NaOH_{(aq)}$, 1 h	153.5 ± 1.0	20.3 ± 1.9	152.2 ± 0.4	23.2 ± 1.6	150.3 ± 0.6	34.0 ± 2.8
Saturated $NaOH_{(aq)}$, 24 h	151.5 ± 0.8	30.1 ± 2.4	143.6 ± 1.3	30.5 ± 1.9	144.4 ± 1.2	45.0 ± 2.8
98% H_2SO_4, 1 h	142.7 ± 0.7	52.3 ± 2.4	149.9 ± 0.8	37.3 ± 2.2	147.3 ± 2.2	44.7 ± 1.8
98% H_2SO_4, 24 h	140.8 ± 1.6	——	137.8 ± 1.9	——	137.0 ± 1.9	——

Supplementary Table S4. $CA_{n\text{-decane}}$ and $SA_{n\text{-decane}}$ of the PAL@fluoroPOS, kaolinite@fluoroPOS and Ca^{2+}-MMT@fluoroPOS coatings after treated under various conditions.
